# Diffusive Public Goods and Coexistence of Cooperators and Cheaters on a 1D Lattice

**DOI:** 10.1371/journal.pone.0100769

**Published:** 2014-07-15

**Authors:** István Scheuring

**Affiliations:** MTA-ELTE Theoretical Biology and Evolutionary Ecology Research Group, Department of Plant Systematics, Ecology and Theoretical Biology, Pázmány P. sétány 1/c H-1117, Budapest, Hungary; Fondazione Edmund Mach, Research and Innovation Centre, Italy

## Abstract

Many populations of cells cooperate through the production of extracellular materials. These materials (enzymes, siderophores) spread by diffusion and can be applied by both the cooperator and cheater (non-producer) cells. In this paper the problem of coexistence of cooperator and cheater cells is studied on a 1D lattice where cooperator cells produce a diffusive material which is beneficial to the individuals according to the local concentration of this public good. The reproduction success of a cell increases linearly with the benefit in the first model version and increases non-linearly (saturates) in the second version. Two types of update rules are considered; either the cooperative cell stops producing material before death (death-production-birth, DpB) or it produces the common material before it is selected to die (production-death-birth, pDB). The empty space is occupied by its neighbors according to their replication rates. By using analytical and numerical methods I have shown that coexistence of the cooperator and cheater cells is possible although atypical in the linear version of this 1D model if either DpB or pDB update rule is assumed. While coexistence is impossible in the non-linear model with pDB update rule, it is one of the typical behaviors in case of the non-linear model with DpB update rule.

## Introduction

The evolutionary stability of cooperation has been in the focus of theoretical biology for decades [1]–[5]. On the one hand, cooperation is widespread and frequent in nature. More importantly in all major transitions of evolution it is connected with cooperation of subunits of the newly evolved replication unit [6]. The spread of cooperative behavior seems to be surprising for the first sight since cooperation is often costly, and cheaters which do not cooperate do not bear the cost of it, and thus can exploit cooperators. Consequently, we might think that cheaters have greater fitness than cooperators, which leads to the extinction of cooperators from the population. Motivated by this discrepancy between field observations and verbal reasoning presented above, many theoretical explanations were given on the origin and evolutionary stability of cooperative act, e.g. [2], [3].

Knowing that the concept of cooperation covers behaviors from extracellular enzyme production of bacteria [7]–[9] through cooperative hunting [10], [11] to eusocial insects [12], it is not surprising that the explanatory mechanisms have great diversity as well. However, alternative explanations are present even within the more narrower areas. For example, there is no consensus as to the main mechanism explaining the evolutionary stability of producing extracellular enzymes (or any other molecules as public good) by microorganisms. One of the widespread explanations is based on slow cell motion and local interactions among densely packed cells. Thus, cooperator and cheater cells distribute in patches. Cooperator cells interact other cooperators (producers) with higher probability than with cheaters (non producers) if their motion is slow and progenies are distributed in the vicinity of the mother cells, so their average fitness will be higher than it would be in a well mixed system. It is shown that if cooperation is not too costly and this assortative pairing is strong enough then cooperator cells can coexist with cheaters even if Prisoner's Dilemma (PD) (the strongest dilemma of cooperation) is considered as the basic model of interaction [3], [13]–[15]. The alternative view emphasizes that local interaction and limited motion lead to positive genetic correlation among interacting individuals, thus kin selection can easily explain the benefit of cooperators [4], [8], [16], [17]. Although these two explanations seem to be only two sides of the same coin, it has been shown recently that they are not always completely identical [12], [18], [19].

However, one certainly important point is generally neglected in the strategic models mentioned above, namely that the produced material, which is a common good for everyone, is frequently a *diffusive molecule*. So, while cells move slowly on the surface they live, the molecules for which competition takes place disperse much faster. More elaborated models should consider this effect. The simplest way to build up this effect into the models is that if the interaction range of individuals is larger than the competition range [20]–[22]. Ifti et al. (2004) used a continuous PD game in a model (individual exhibits variable investment into cooperation) where individuals live on a 2D rectangular lattice. They found that if difference between interaction range and competition range exceeds a critical level then cooperation is not sustained [20]. Increasing interaction range is not so dramatically detrimental to cooperators in a nonlinear public goods game on a two dimensional lattice, where the common benefit is a sigmoid function of the produced material within the diffusion range. It is shown that producer and non-producer can coexist even if the diffusion range is much larger than interaction range, although the fraction of producers decreases with diffusion range [22]. Interestingly in a spatial model of self replicating macromolecules which cooperate in maintaining a metabolic cycle and compete for the same sort of monomers at the same time, some difference between interaction and competition range is necessary for the coexistence of replicator molecules and for the decline of cheating parasites [21], [23].

A recent paper by Allen et al. (2013) studied this problem in a model where public good units move randomly along a graph [24]. They studied the probability of fixations of rare producer and non-producer mutants in a case when diffusive public good is in a linear relation with the fitness of strategies. They defined the producer cells to be evolutionary supported if the fixation probability of a single producer among the non-producers is greater than the fixation probability of a non-producer among producers. They found for a wide range of graphs (including 1D and 2D rectangular lattices as well) that producers are evolutionarily supported if the benefit remains at the producer and the total benefit retained by the neighbors of it is greater than the cost of production of the public good [24].

Another recent paper by Borenstein et al. (2013) have studied a 2D lattice model in which cooperator cells produce a diffusive material which is a common good and fitness is a (linear or saturating) function of the concentration of diffusive material. The model assumes that every lattice point is occupied by a cell. They use a birth-death algorithm: progeny of the neighbors of a randomly chosen cell replaces this cell proportional to their relative fitness. Cells die according to an exponential process inversely related to the fitness of the cell. They concluded that cooperators cannot coexist with the defectors in this model. This does happen even if fitness saturates with the concentration of diffusive material [25]. These results are surprising first because of the experience of coexistence of cooperators and cheaters in spatial models of cooperation (see above), second because coexistence of cooperators and cheaters is typical if public good is a saturating function of the effort even in well mixed [26]–[30] and similarly in spatial models [22], [31].

Borenstein et al'.s (2013) 2D model is too complex for analytical investigations, thus their conclusions are based mainly on numerical simulations [25]. However, 1D models can more frequently be analyzed mathematically which helps to understand the dynamics of the 2D model and/or to reveal the connection between model details and dynamics. For example the connection of dimensionality with dynamics is an important question since biofilms can change from 1D dendrite line to a rather 3D shape, although they are generally a fractal [32], [33]. Further, the dynamics of spatially explicit models generally depends on the order of elementary steps, that is, whether birth-death or death-birth update rules are applied [34]–[38], and whether interactions modify fecundity or/and survival [39].

Here I focus on a 1D system similar in some properties to Borenstein et al.'s 2D model. In the following sections I analyze the dynamics of this system mathematically and numerically as well by using two types of death-birth update rules. The first rule assumes that a cell stops to produce common material before death, thus concentration distribution is computed without this producer. This rule is termed as death-production-birth and is denoted by DpB. The second rule assumes that a producer cell synthesizes the common material before it dies, so the concentration distribution of the diffusive material computed before this death event, and birth success depend on this concentration. So this will be called the production-death-birth rule denoted by pDB. I have shown that independently to the used update rule coexistence is possible in the linear model (fitness increases linearly with local concentration of the common material) although this behavior is rather atypical. However, coexistence is a robust behavior in non-linear model (if fitness is a saturating function of local concentration of the common material) if the pDB update rule is used while coexistence is impossible if the DpB update rule is applied. In the last section, I compare them with results of previous similar models of bacterial cooperation.

## Analysis

Consider an 1D lattice of lattice size 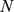 where every lattice point is occupied by a 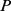 (producer) or an 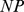 (non-producer) cell. The grid is 1-dimensional, thus every cell has two nearest neighbors except the ends of the lattice where cells have only one neighbor. I choose this boundary condition, since it follows the biological situation and makes the calculations simpler. 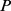 cells are considered to be point sources of diffusive materials which is a public good for every cell in the lattice. The diffusive material decays with a constant rate. Hence the concentration field 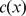 of a single 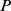 cell at 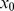 is determined by
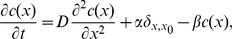
(1)where 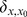 is the Dirac delta which sets the position of the 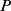 cell at 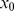, 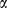 is the rate of production of the material, and 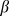 is the decay rate of it [25]. Since diffusion is much faster than replication I may consider the steady state solution of (1), that is if 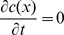 [25]. Then the solution of (1) is
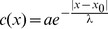
(2)where 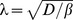 is the diffusion length, 
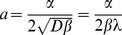
 is the steady state concentration at the source, 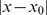 is the distance from the source [40]. Naturally, if there are more than one 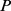 cells on the lattice then 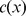 can be computed as the superposition of concentration fields of 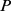 cells
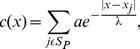
(3)where 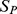 is the set of indices on the lattice containing 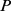 cells.

**Figure 1:**
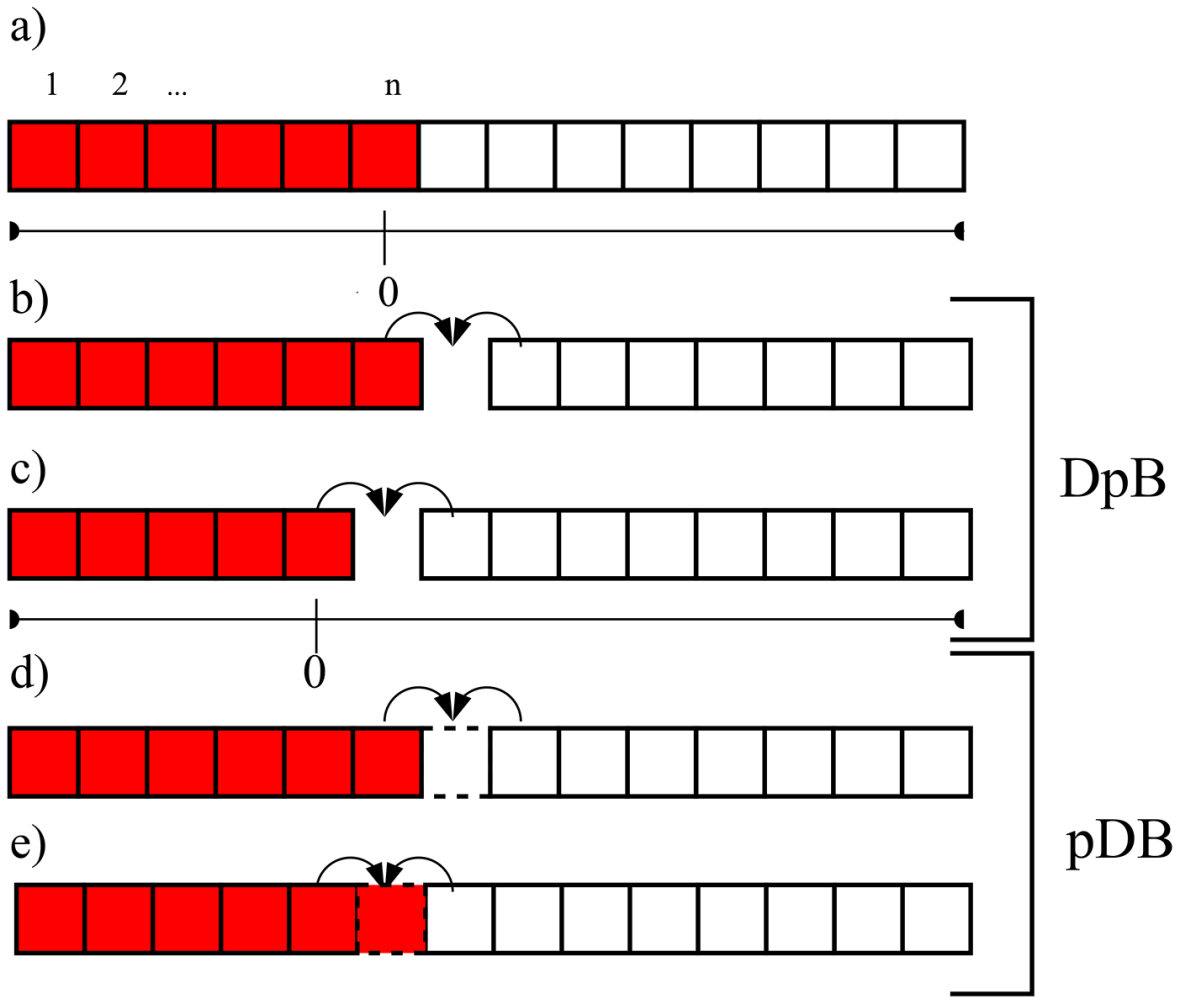
The initial distributions of 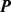 (red) and 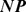 (white) cells with a 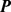 array being 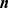 cell long (a) and the possible invasion events at the border of arrays if DpB update rule (b, c) and if pDB update rule is assumed (d, e). For DpB update rule first a cell dies, then the concentration of the diffusive material is computed and finally reproduction occurs, for pDB update rule first the concentration of the diffusive material is calculated, then reproduction and finally death take place. Arrows indicate the nearest neighbors competing for the empty site. Origin is placed at the 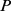 cell being at the actual border of 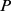 and 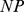 arrays as indicated in figure (c).

It is assumed that fitness differs in the replication rate of individuals which depends on the local concentration of the diffusive material and all cells take up this material with the same efficiency. Since 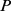 cells produce a costly material, their replication rate is decreased by a constant factor. Mixing of cells is limited along the lattice in this model, consequently a cell can place progenies only to the neighboring site. Update occurs according to either a DpB or a pDB process (Fig. 1). For DpB process, a cell is chosen randomly to die on the lattice and the concentration of the diffusive material and replication rates are computed after that in the neighborhood of this empty site. Then, one of the neighbors' progeny will occupy the empty cell proportional to its relative fitness [14], [34], [36], [37]. For pDB update rule, concentration distribution of diffusive material and replication rates of cells are computed first, then a cell is selected randomly to die. This empty grid point is occupied by one of the neighbors according to their relative replication rates. (That is, the selected cell dies after the birth of progenies, and if this cell was a P cell, then its produced common material must be involved in the calculation.) More details of the algorithm, for example how fitness is calculated is described in the following. Progenies follow a strategy identical to their adults, and there are no mutations or recombinations.

### Initial condition I: two different arrays of cells

First, I study the case when an array of 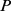 cells meets another array of 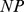 cells. Here I study competition of monomorph arrays for the space, which does occur when two growing 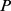 and 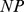 arrays meet. (The growth of arrays before meeting is without competition, thus the dynamics before meeting is of no interest to me.) I place the origin at the border where 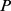 and 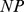 arrays are in contact (so the origin moves along the moving front). Without loss of generality I assume that 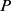 cells are left to the origin, and 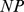-s are right of it (Fig. 1 a.)

Since replication and decay events change the configuration only if it occurs on the border of 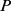 and 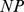 cells, I am interested in the 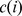 concentration in the neighborhood of the border. If the number of 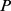 cells is 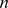 in the 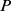 cell array then the concentration of the diffusive material within the 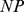 array at the coordinate 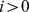 from the border is
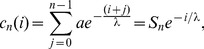
(4)where
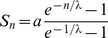
(5)is the sum of a geometric series.

### Replication rate depends linearly on concentration

In this section, I study the case when replication rate 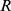 of a cell is a linear function of the the concentration of the diffusive material within the cell, that is
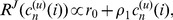
(6)where 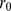 and 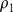 are positive constants, 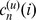 is the concentration of the uptaken material in the cell. 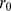 is the basic replication rate independent of the cell type, while 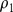 scales the concentration of diffusive material into replication success, 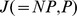 denotes one of the two cell types. I assume that the time scale of uptake of diffusive material is much longer than the diffusion time scale, thus the uptaken material is proportional to local steady state concentration, that is 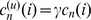, where 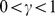. Therefore 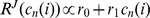 with 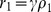. Since every site is occupied by a cell, and every cell consumes the material with the same rate, it follows that the uptake of diffusive material only rescales the steady state concentration outside of the cells, that is 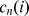 decreases to 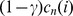. Producing the diffusive material is costly and it decreases the replication rate of 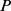 cells with 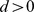. Since cell types can change only on the border of 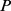 and 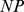 arrays, I am interested in the replication rate of 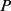 and 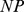 cells in the neighborhood of the border.

By using DpB update rule the size of 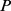
's and 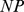
's array can change if either the 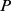 or if the 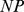 cell dies on the border, and the other cell type occupies the empty site (Fig. 1 b,c.). The replication rate of 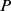 on the border is
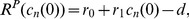
(7)while the fitness of the second neighbor 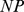 cells of this 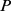 is
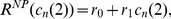
(8)where 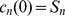 and 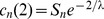. It is clear that if 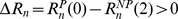 then the 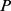 array increases with a cell with higher probability than the probability of remaining the same size (Fig. 1 b.). Based on this observation, I use the following definitions:

#### Spreading


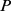
 *spreads* at state 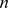 if 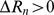. If 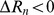 then the 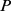 array remains the same with higher probability than to increase with a 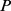 cell, that is 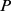 *does not spread*. Similarly if 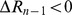 then 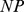 spreads on the 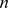 array of 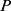-s, and if 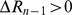 then 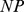 does not spread (Fig. 1).

#### Stability

A state of 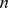 array of 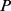-s is defined to be *locally stable* if 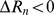, and 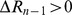, that is neither 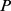 nor 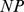 spreads at this state, and the 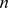 array of 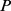-s is *locally unstable* if 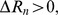 and 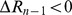, that is both 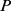 and 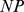 can spread. Naturally, if fitness differences are zero (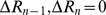) then the state is *neutrally stable*).

By using the definition of stability introduced above, I can formulate lemmas which help the analysis.

#### Lemma 1

By using DpB if 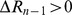 then 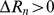 for every 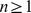, that is, if 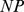 doesn't spread then 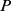 spreads at state 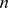.

Similarly, if 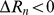 then 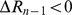 for every 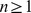, that is if 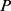 does not spread then 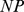 spreads at state 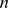.

#### Proof

According to (4,7,8)
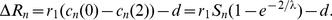
(9)


The lemma follows from the strict monotonicity of 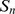 in (9).

The corollary of this lemma is **Lemma 2**:

By using DpB if 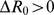 then 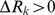 for every 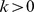. That is if a single 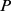 spreads, then it always spreads and fixates in the population with higher probability than it would be in the neutral case.

Similarly, if 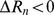 then 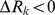 for every 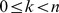. That is if an 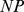 cell can invade an 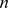 array of 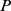-s then it invades every smaller array of 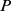-s. Thus, 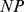 fixates in the population with a higher probability than it would be in the neutral case.

The direct consequence of Lemma 1 and Lemma 2 is that if one of the cell types can spread on the border of the other cell array it always spreads until fixation. The remaining case is when 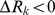 if 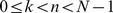, but 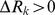 for 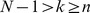. Knowing that 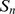 increases strictly monotonously with n this situation is possible (see 5,9). Then state 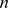 is an unstable equilibrium of 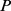 and 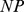 arrays from which either 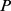 or 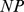 spread and fixates.

If pDB update rule is assumed, then conditions of spreading of 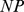 and 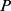 have to be considered separately. Similarly to the DpB process, 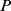 does not spread if 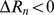, and spreads if 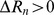 (NP cell is deleted, compare Fig. 1 b and d). However, if the 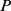 cell being on the border of 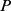 and 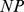 blocks is replaced by a new progeny by one of its neighbors (Fig 1 e), then the order of computing the common material and deleting the P cell make difference. Here fitness of the neighboring 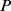 and 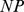 cells will be
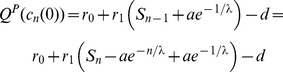
(10)and
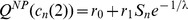
(11)respectively. (Replication rates are denoted by 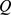 to highlight the difference between DpB and pDB updates.) 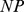 does not spread if
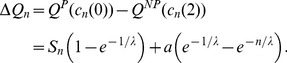
(12)


Lemma 2 is valid for 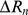, that is if a single 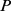 spreads (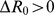) then 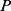 always spreads and fixates. By using pDB update rule a similar lemma can be formulated for 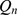.

#### Lemma 3

If there is an 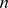 for which 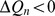 then 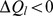 for every 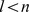.

#### Proof


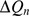
 is a strictly monotonically increasing function of 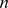 since 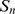 and 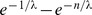 increase strictly monotonously.

It follows from Lemma 3, that if a single 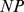 spreads (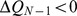), then 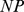 always spreads and fixates in the population. Further, the direct consequence of Lemma 2 and Lemma 3 is that if a single cell can invade the population of other cell type, then it always spreads and fixates. There is a locally stable polymorph state if 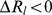 and 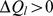 for 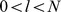. However, the following lemma excludes this possibility:

#### Lemma 4


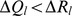
 for every 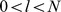.

#### Proof

Using 9 and 12 and the expression of 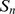, after simplifications we obtain the following relation:
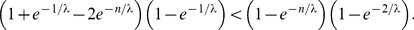
(13)which simplifies to 
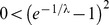
. That is, Lemma 4 is valid and, consequently, stable polymorphism is impossible in case of pDB update rule as well. As in the case of DpB update, it follows from lemmas 2–4 that an unstable polymorph state is possible.

Summarizing the results: Assuming that replication rate is a linear function of the local concentration of a diffusive material and by using the simplest geometry of arrays in 1D lattice (that is there are two arrays of cells) I have shown that stochastic coexistence is impossible independently of the applied update rule. Depending on the parameters either 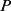 or 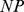 cells spread and fixate in the population or there is an unstable state of coexistence which divide the dynamics into two attractors; then depending on the initial condition either 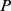 or 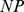 fixates in the population.

### Replication rate depends non-linearly on concentration

In this section, I study the dynamics of the system when 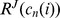 is a nonlinear function of 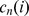. Motivated by the Michaelis-Menten kinetics, I consider the following function of reproductive rate as it depends on local concentrations:
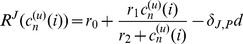
(14)where the role of 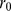 and 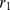 are the same as before, while 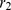 gives the concentration where 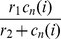 reaches the half of its maximal value (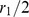), 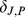 is the Dirac delta (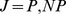). Biologically, a function of this form could arise in two different ways. Either the enzymatic effect of the diffusive material within the cell limits the speed of the dynamics or the uptake of the material is the limiting step. Again I assume that the time scale of uptake is much longer than the diffusion time scale, thus the uptaken material is proportional to local steady state concentration. In the first case, uptake rate is proportional to the local extracellular concentration. Then, the enzymatic effect of the diffusive material is saturated within the cell. In the second case, uptake rate is saturated as the concentration of the diffusive material increases. Thus in the first case 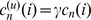 as in the linear model, but 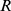 depends nonlinearly on 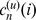. Because of linear relation between 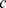 and 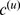


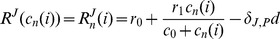
(15)with 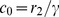. Since uptake is linear and assumed to be much slower than the production-decay dynamics of the diffusive material, it only rescales the steady state concentration as in the linear case above.

In the second case 
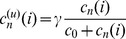
 and 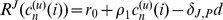. These relations give back (15) with 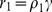 and 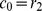. The difference between the two cases in the way they modify the local concentration of the diffusive material outside the cells. Again, if production and spontaneous decay are much faster than uptake, then 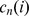 modifies to 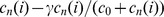 which is a good approximation in the steady state.

As before, I am interested in the fitness difference of 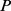 and 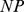 cells on the border of arrays. By using DpB update rule merely the 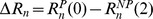 relation has to be studied again. While 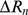 is a strictly increasing function of 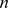 in the linear model (see 9), this is not necessarily the case in the saturating fitness function. It is easy to show that if 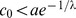 then 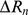 is a monotonically decreasing function of 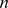. So if 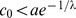, then the following dynamics are possible:

1. If 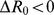 then 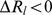 for every 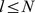. Consequently, 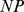 always spreads and fixates in the population.
2. If 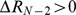 then 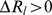 for every 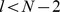. In this case, 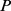 always invades and fixates in the population.
3. If 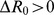 and there is a 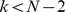 where 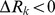. Then 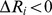 for every 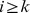 and 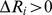 for 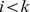. Thus 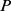 spreads until the array of 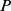-s attains size 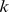 and does not spread further from this state since 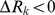. Similarly, 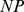 spreads if the 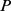 array is larger than 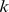. Since 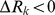 and 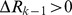, neither 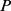 nor 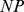 spreads in the 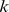 array of 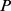-s. Consequently, 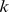 array of 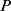 is a stable state of 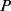-s and 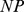-s.

The situation is different if pDB update rule is applied. According to Lemma 4, coexistence is possible if there are parameters such that 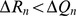, that is if
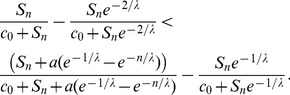
(16)


However, this is not possible.

Using the defined saturating fitness function, it is clear that
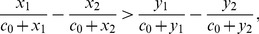
(17)if 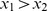, 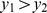, 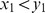, 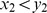 and 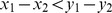. In the present case, it is easy to show that 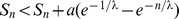, 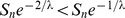 and 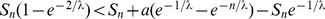, consequently 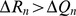. Thus, coexistence is impossible even if a non-linear saturating fitness function is assumed.

### Initial condition II: 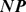 emerges by mutation

**Figure 2:**
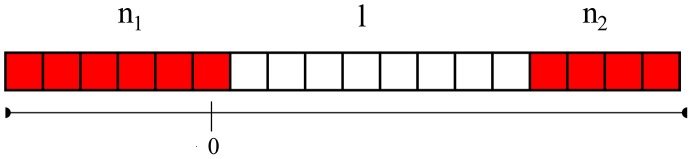
The geometry when an 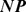 array (white) emerges by mutation within the 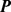-s (red). 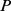
 arrays contain 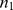 and 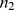 number of cells while 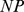 array is 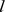 number of cells long.

Another biologically relevant case is when the population initially consists only of 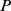 cells. Then a 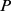 cell mutates into an 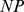 cell and this 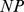 cell starts to spread. This mutation event can happen everywhere within the 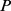 array, and to model this situation I consider the initial geometry when two arrays of 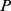 cells straddle an array of 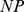 cells. I place the origin at 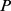 cell which contacts an 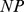 cell from right (Fig. 2) and I compute the concentration of the common material in the neighborhood of this meeting point.

By using DpB update rule the concentration is
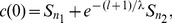
(18)at the origin, where 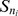 (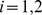) is the sum of concentrations arriving from the 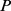 cell arrays of length 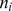. Since the length of the 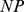 array is 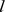, thus the concentration of the material from the 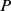 cells right to the origin decreases with 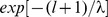 (see Fig. 2). Similarly the concentration at the 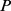 cell which has 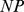 cells to the right is
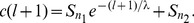
(19)


The 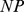 cells being the second nearest neighbors of 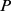 cells on the left and right end of the 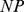 block feel concentrations
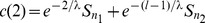
(20)

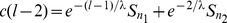
(21)respectively. Following the analysis made before the local stability of a state is studied. I compute the concentrations in the neighborhood of the borders of arrays after the 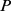 cell being on the border is died. Then, the nearest neighbor 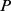 cells feel concentrations
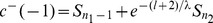
(22)

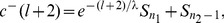
(23)where 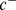 denotes that the 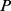 cell is died on the border. The first relation refers to the situation when 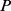 dies on the left border and the second relation refers to the situation when 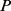 is deleted on the right border. Similarly, the nearest neighbor 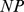 cells of the empty sites feel concentrations
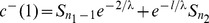
(24)

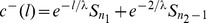
(25)on the right and the left borders, respectively.

Assuming again that replication rates depend linearly on local concentrations (see 3) neither 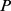 nor 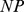 spreads on the left border if
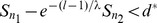


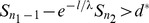
(26)and similarly neither 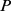 nor 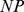 spreads on the right border if
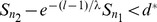


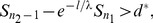
(27)where 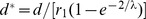.

To find coexistence of 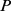 and 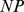, all relations of (26) and (27) must be valid simultaneously. After studying these relations, it turns out that there can be locally stable states 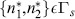 and unstable states 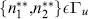 at this initial condition (for details, see Text S1), that is, coexistence is possible in this case, while the graphical representation of relations (26) and (27) is depicted in Figure S1.

Alternatively, if pDB update rule is applied and the same computation is used as above then relations (26) and (27) are replaced by
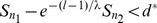


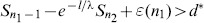
(28)and
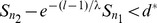


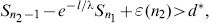
(29)where 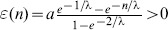 for every 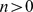. Following the same calculation as presented in text S1, it is easy to see that including 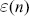 into the relations does not change the general argumentation. Consequently, there can be a locally stable and an unstable state where 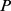 and 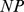 cells are in coexistence even if pDB update rule is used.

### Nonlinear case

The method described in the previous section is not applicable in this case. However, I am mainly interested in the possibility of coexistence, which can be studied without any further calculation, at least in the DpB case. We can have four characteristically different dynamics if an 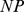 array is inserted initially into the 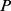 array:

1. There is a state where an 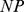 array coexists between two 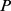 arrays (neither strategy spreads at the borders). According to the analysis of the linear case, it is possible (e.g. if fitness is almost linear), but I could not show it for a general saturating fitness function. Thus, this situation is not considered as a case of coexistence.
2. The second possibility is when 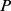 always spreads and 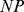 does not spread at both meeting points. Then 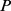 wins over 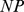. There is no coexistence.
3. Similarly, if 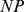 always spreads and 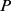 does not spread at both meeting points, then 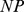 wins over 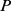.
4. However, if 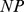 spreads at both meeting points, then 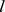 increases, and it is possible that for a sufficiently high 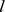 the effect of the remote 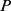 block (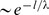) can be neglected. Thus, pairs of 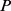 and 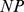 arrays can be studied separately. But I have shown that if 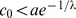 then coexistence is possible.

So without analyzing the nonlinear case mathematically at this initial condition I have shown that coexistence is possible if DpB update rule is used because dynamics may lead to a distribution of 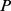 and 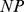 arrays for which the possibility of coexistence has been proven.

The situation is different if pDB update rule is assumed. Then, as I have shown earlier coexistence is not possible if pairs of 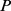 and 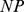 arrays can be studied separately. Thus the above described argumentation is invalid here. It is possible however that balancing effect of two 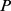 arrays can maintain stable coexistence as in the linear case (it must be true at weakly non-linear fitness). This possibility is studied by numerical simulations in the following section.

### Coexistence and the position of 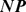 array: numerical simulations

I have shown earlier that 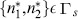 is a locally stable state in the linear model. However, the analysis could not reveal completely the direction of motion in the 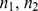 phase space, although the analysis provided some information about it (see Text S1). Even this information is missing in the nonlinear case. Thus, the dynamics is studied by numerical simulations with DpB and pDB update rules as well.

I consider a 1D lattice of size 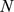 where every lattice point is occupied at most by a cell. 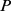 cells are considered as sources of the diffusive material. I use the following algorithm to simulate the DpB update rule:

1. A cell is deleted at a randomly selected lattice point.
2. The concentration 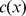 is computed according to (3).
3. Replication rates (fitness) of the cells are calculated from the local concentration of the diffusive material according to linear or nonlinear relations defined above.
4. The empty cell is occupied by the copy of those neighboring cells which have a higher replication rate.
5. A new cycle begins.

The algorithm of pDB update rule is very similar except that steps 1. and 2. are interchanged in the series presented above. This update cycle is repeated until one of the strategies dies out or the coexistence of 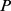 and 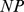 cells is observed for a long time. I use deterministic update rule just to fit the algorithm of the numerical simulations to the analytical studies. Other rules e.g. [14] considering probability of success as a function of relative fitness leads qualitatively the same results if selection is not too weak.

**Figure 3:**
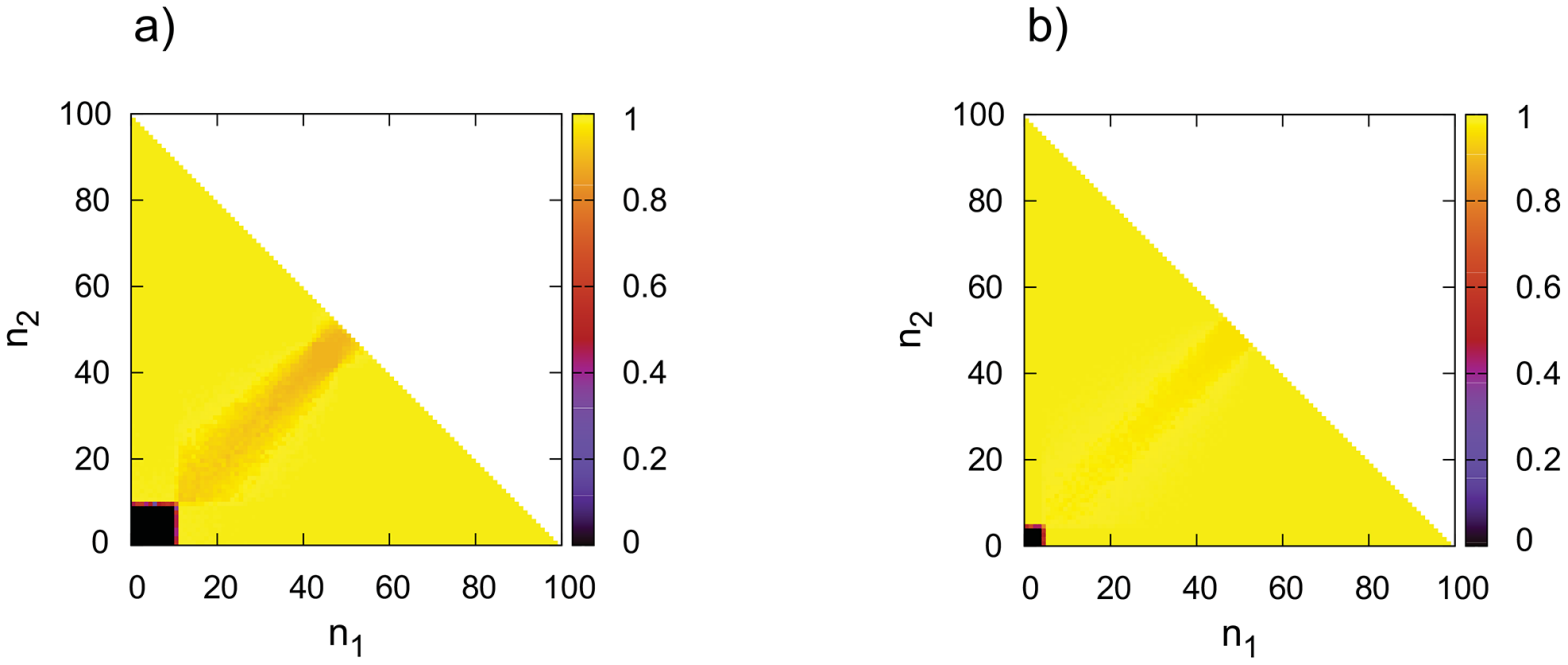
Dynamics of the linear model in function of initial sizes of 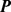 blocks (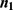 and 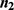 respectively). Colors reflect to the frequency of 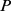 cells in the equilibrium (darker means less fraction of 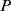 cells). **a**) DpB update rule, the cost of producing diffusive material is higher 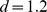, **b**) DpB update rule, the cost of producing diffusive material is lower, 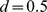. Other parameters are 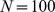, 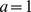, 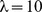. The depicted values are averages of 50 independent simulations. By using pDB update rules gives practically the same results, so they are not showed.

**Figure 4:**
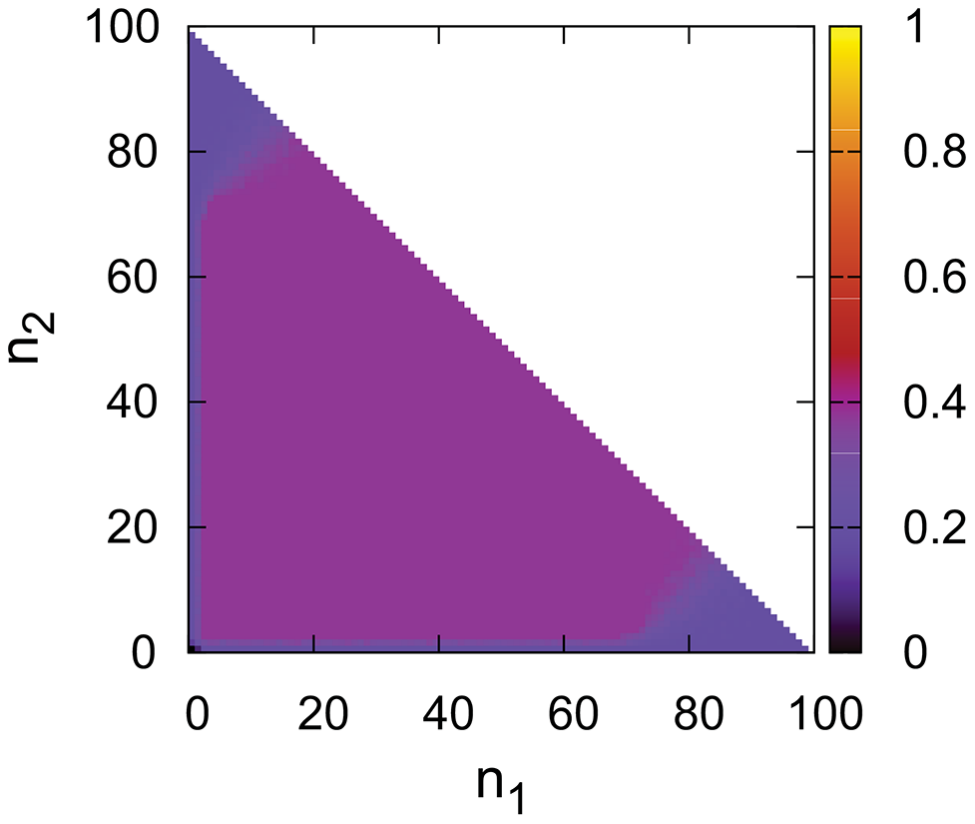
Dynamics of the nonlinear model with DpB update rule in function of initial sizes of 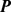 blocks (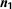 and 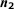 respectively). 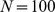
, 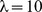, 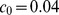, 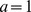, 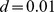. Values are averages of 50 independent simulations.

By using a wide range of parameters of 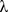 and 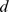, I observe that if coexistence between 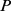 and 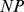 is possible in the linear model, then this state will be realized only if the inner 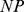 block is close to the center of the lattice independently whether DpB or pDB update rule is used, that is if 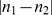 is less than a critical value (Fig. 3). Otherwise, either 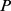 or 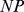 fixates in the population. If update rule is stochastic then the system will be fixed fast in one of the monomorph states independently of the initial condition, since the population can easily move away from the narrow basin of attraction of the stable state where 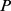 and 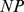 are in coexistence. The situation is different in the nonlinear case. Then coexistence is realized independently from the initial condition, only the fraction of 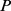 cells in the equilibrium depends on the initial values of 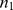 and 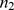 (Fig. 4) if DpB update rule is used. However, I have not experienced coexistence of strategies for a wide parameter range if pDB update rule is used (except for the cases when the non-linear function becomes practically linear).

### Randomly placed cells: numerical simulations

In this section, I consider the case when 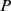 and 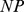 cells are initially placed along the lattice at random. I fill up every lattice point with 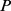 cells with probability 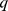 and with 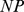 cells with probability 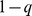. The above described analysis would even be more complex in this case, thus I apply numerical simulations to reveal the dynamics.

**Figure 5:**
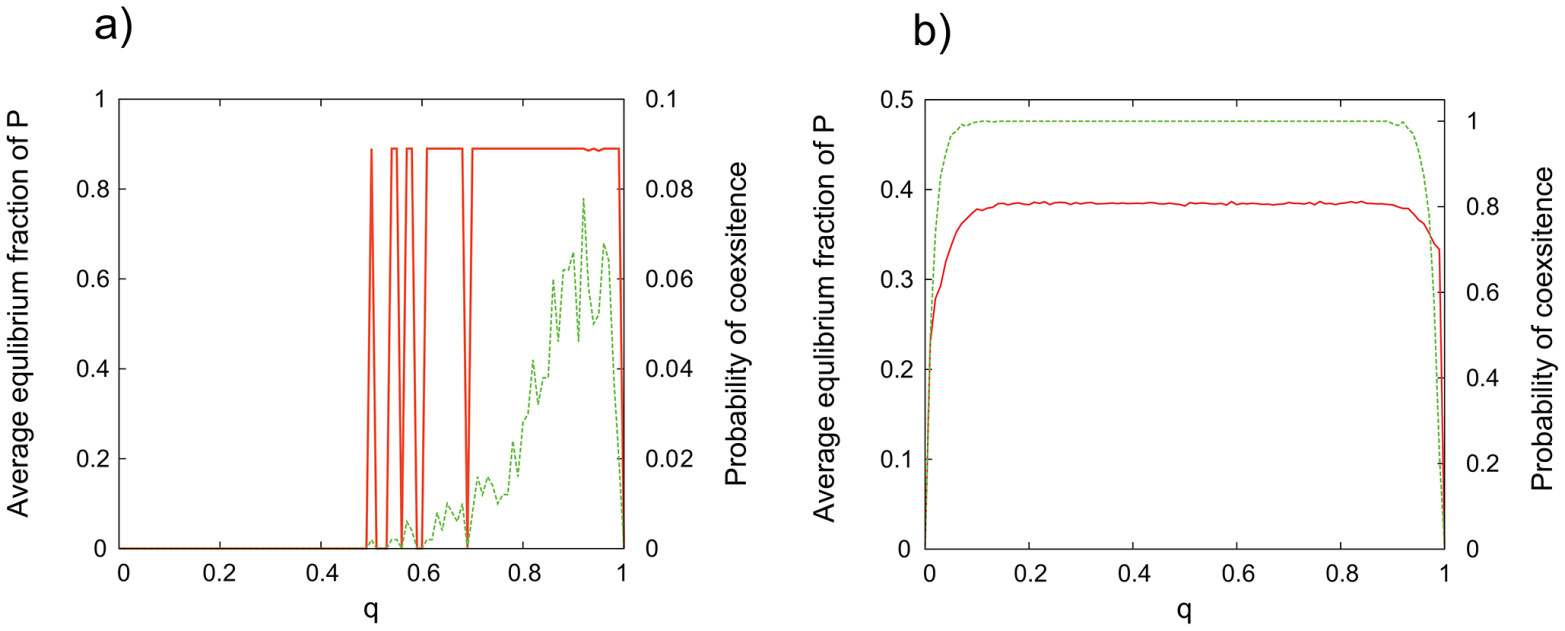
Average fraction of 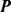 cells (red, solid line) and the estimated probability of coexistence in function of the initial ratio of 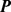-s (green, dashed line) if 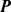 cells are randomly placed initially. Red lines denote the average fraction of 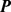, green lines denote the probability of coexistence. **a**) Linear model, DpB update. 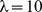, 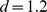. (The model with pDB update rule behaves practically identically at these parameters.) **b**) Nonlinear model, DpB update. 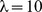, 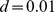, 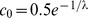. Plotted values are averages of 500 independent simulations, 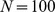, 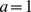 in all simulations.

**Figure 6:**
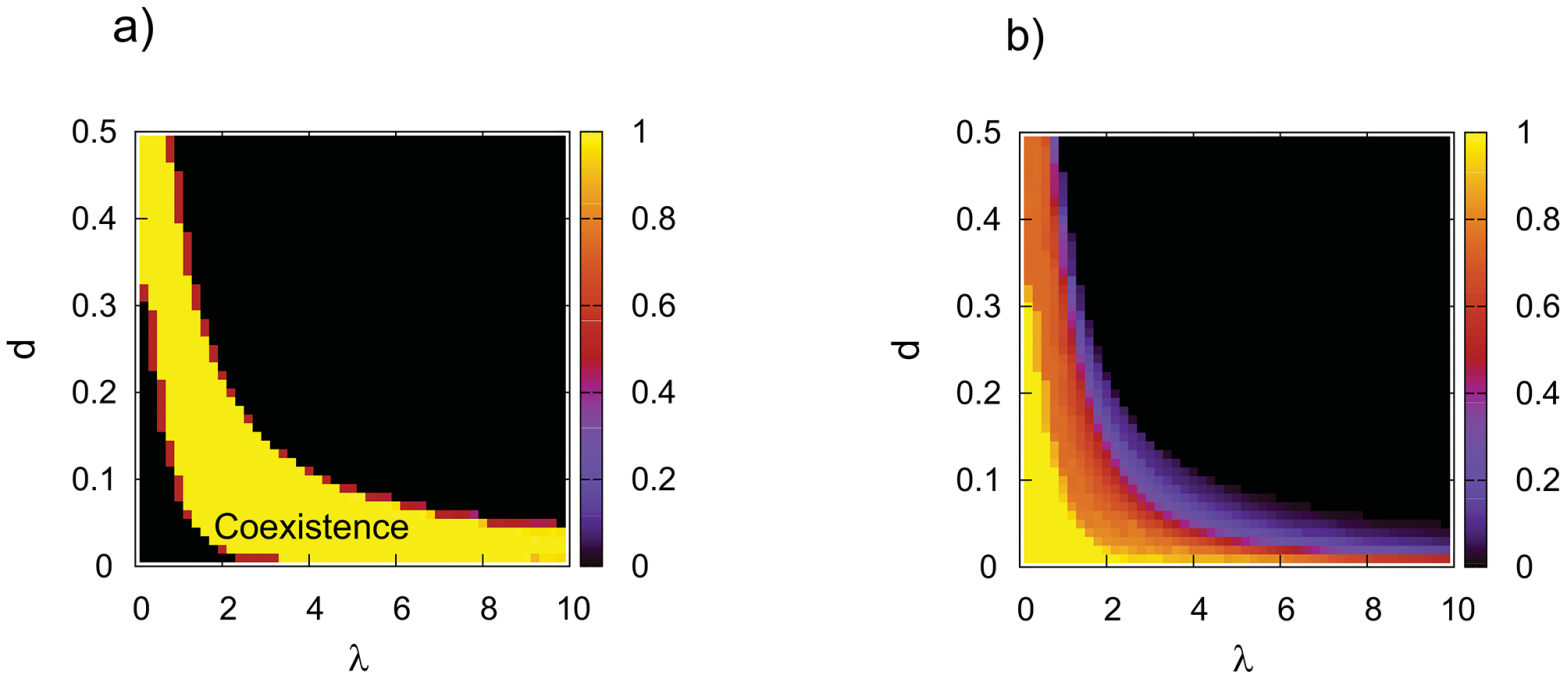
The qualitative dynamics of the nonlinear model with DpB update rule in function of the diffusion length (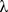) and the cost of producing diffusive material (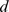). **a**) The average probability of coexistence. **b**) The average frequency of 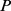 in equilibrium. Values are averages of 50 independent simulations, initially 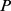 and 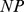 cells are distributed randomly with the same probability, 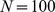, 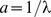, 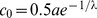.

I used the same algorithms (DpB and pDB update rules) and the same parameter values as in the previous section, but here the dynamics is studied in function of 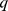. Considering the linear model I observe again that the coexistence of 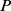 and 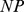 cells is not robust, and can be observed only if 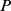-s are present initially with a sufficiently high probability (Fig. 5). In concordance with results presented above, coexistence was not observed for nonlinear model with pDB update rule even if 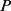 cells were placed randomly along the lattice. On the other side, coexistence remains a robust behavior even if random initial condition is used in the nonlinear model with DpB update (Fig. 5 b). Based on the fact that dynamics of the nonlinear model with DpB update rule is not sensitive to the initial condition (see Fig. 4, 5 b) an arbitrary random initial distribution can be selected and the effect of dynamical parameters on the coexistence can be studied in this case. Probability of coexistence and the average equilibrium frequency of 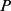 cells are measured in function of the cost of producing of diffusive material (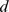) and diffusion length (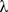). Based on the simulations it is clear that strategies can coexist in a wide range of parameters (Fig. 6) although fitness differences can be very small (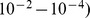) in the studied parameter space.

## Discussion

I studied the 1D version of Borenstein et al.'s (2013) model with linear and non-linear (saturating) fitness function by using two different types of death-birth update rules. It is shown that independently of the used update rule coexistence of producers and non-producers is impossible in the linear model if initially a producer and a non-producer arrays meet on the lattice. On the other hand I have shown that coexistence of strategies is possible for the non-linear model by using this initial distribution of strategies with DpB update rule, but impossible if pDB update rule is considered. Furthermore, I have shown that if a non-producer array is inserted between two producer arrays, then coexistence of cell types is possible even in the linear model for both update rules. Numerical simulations verified this result but revealed that basin of attraction of this stable state is narrow, that is dynamics leads to this stable state only from very specific initial states. On the contrary coexistence is non-sensitive to the initial condition for non-linear model with DpB update rule. It is even more striking that coexistence is observed at a wide parameter range of diffusion length and cooperation cost for this model. To sum it up, coexistence of producers and non-producers is a non-robust phenomenon in the linear model with both update rules, a typical phenomenon in the non-linear model with DpB update rule. However, coexistence is not observed in the non-linear model with pDB update rule. The observation that update rules modify the result of selection is not new. It has been shown several times that death-birth process promotes cooperators in structured populations [34]–[37], and well known that details of the spatial model greatly modifies its behavior [41]. The difference here is that two alternative death-birth processes can be defined, and saturating fitness function leads to the robust coexistence of producer and non-producer cells only of the DpB rule is applied.

Borenstein et al. (2013) have not studied the effect of initial distribution of cell types on the long term dynamics and they focused on the model with a birth-death update rule. They used a stochastic update rule, where the success of the competing cells are proportional to the growth rates of competitors. In the numerical simulations I used a model where competitor with higher replication rate always wins over the competitor with lower replication rate. Further, they used the standard periodic boundary condition in the simulations, while I defined a model with open ends. So their model differs from mine in many crucial details, so that, the direct comparison would have only a limited validity. Because the fitness differences are small for most of the selected parameters, the deterministic update rule speeds up the simulations significantly and helps to check the analytical results more easily. According to numerical simulations, if population size is not too small and selection is strong, although update is not deterministic, then the deterministic and stochastic models behave very similarly (not shown). This is generally not true for 2D systems where stochastic and deterministic update rules generate different 2-dimensional patterns which have long range spatial effect and modify the dynamics significantly [42]. The 1-dimensional model is much simpler in this sense, stochastic and deterministic update rules develop the same spatial patterns.

I have shown that initial condition and update rule can have significant effect on the dynamics of the 1D model with non-periodic boundary condition. Coexistence is not robust in the linear model, it is possible at specific initial conditions. The intuitive reason of the non-robustness of coexistence is that if the spread of either 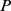 or 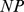 is favored then spread is more favored if their number increases (see Lemmas 1–4). Only very specific initial geometry can smooth this robust effect. The situation is characteristically different if replication rate depends nonlinearly on the common material. Coexistence is a typical and robust behavior if DpB update rule is applied, while coexistence is impossible if pDB is used. Interestingly, the general theoretical [26], [27], [30] and experimental [7], [43] results support that saturating effect of public good on fitness can stabilize coexistence of producer and non-producer cell types even in well-mixed models. Although I presented a spatially structured model now, where individuals interact only with their neighbors, diffusion of limiting resource smooths out local differences, and thus resembles in a sense to models of well-mixed populations. On the other hand, the diffusion and saturating effect on the fitness smooth out the differences among individuals, thus relative fitness differences are generally small, which means that while stable coexistence is typical, it is generally closely neutral and leads to high fluctuations in frequencies of cell types.

As I mentioned in the Introduction, Allen et al. (2013) studied the probability of fixations of rare producer and non-producer mutants for a wide range of graphs (including 1D and 2D rectangular lattices as well). (Their work does not examine the possibility of coexistence. In a finite stochastic population one of the strategies fixates sooner or later. However, fixation time increases polynomially or exponentially with population size if there is a stochastically stable polymorph state, so coexistence (as a long term metastable state) is practically present for larger populations [44]). They showed that producers are evolutionarily supported if the benefit remains at the producer plus the total benefit retained by the neighbors of it is greater than the cost of production of the public good [24].

Considering the linear version I receive a similar relation in the model. From the calculations above it is clear that a single producer cell will spread and fixate in the population if the benefit retained by the cell minus the benefit arriving at the nearest non-producing cell is greater than the cost of cooperation, that is
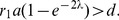
(30)


(This relation comes from rearranging 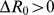 in (9).) In agreement with the intuition, if 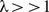 (public good is distributed evenly) then the maximum benefit should be very high (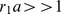) to support producers. Contrariwise, if 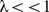 (good is private) then public good is supported if private benefit is larger than the cost of producing it (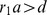). Another recent study show that extended durability of public goods reduces the selective advantage of cheater strategy if public good production is regulated according to the concentration of the diffusive material [45]. In the present model, producing more durable public goods means that parameter 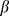 becomes smaller. To explain the previous relation in function of diffusion constant and spontaneous decay of diffusive material 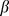 one receive
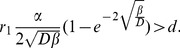
(31)


It is clear that if the diffusion rate is constant and public good is more durable (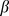 decreases) then the relation is more easily satisfied (right hand side increases). If 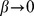 then 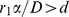 gives the condition for the cooperators to spread and fixate, that is the production speed and the diffusion speed of the material determine the success of producers.

Earlier studies estimated 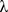 to be on order of one (roughly between 1 and 10) in experimental situations [24], [25]. The numerical simulations support that coexistence of producers and non-producers is possible in this region, mainly for the non-linear model with death-birth update rule if the cost of producing this material is not too high (see Fig. 3.,6.). Interestingly, Julou et al. (2013) have shown experimentally that 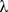 can even be much smaller in bacterial colonies growing on solid phase. Here mainly the local exchange of material among contacting cells determines the dynamics.

My simplified model neglects numerous details which are present in real biofilms. For example bacteria grow on a surface on the border of liquid and solid phases. They can be in competition for space, for limiting resources on the surface and for 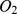 arriving from the water [8], [46]. Furthermore, bacteria communicate each other by quorum sensing, and collective action is not only production of common material but they can change their motion strategy as well according to local environmental and social circumstances [47]–[50]. It is useful to compare the results of these complex models with my results regarding the coexistence of cooperators and cheaters. Xavier and Foster (2007) studied competition for the soluted 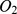 in a biofilm by a specific individual based model [46]. Producer cells extract extracellular polymeric substances (EPS) which is assumed to help to maintain the structure of the biofilm. According to the simulations, the EPS producing cells outgrow the cheater cells and reach higher concentration of diffusive 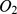, which is a growth limiting factor in the system. Depending on the parameters and initial conditions either producers or cheaters win the competition, but coexistence is observed in certain cases. Unfortunately, this situation is not studied in detail. On the other hand a more recent study on this system does not mention the possibility of coexistence [51], but emphasizes that depending on nutrient availability and diffusion selection may result in 1) either spatial segregation of lineages and spread of producers or 2) mixing of types and winning of non-producers.

## Supporting Information

Figure S1
**Graphical representation of stable and unstable meeting points of** 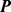 **and** 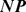 **cells in function of** 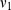 **and** 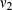 **.** Solid lines denote the stable while dashed lines denote the unstable states. **a)** The complete phase space. Arrows represent the motion qualitatively. Rectangles around the intersections are magnified in figure b) and c). **b)** The locally stable state. 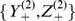, 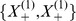, 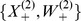 and 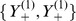 are the intersections of 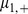, 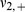, 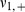 and 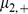 which determine 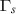. **c)** The locally unstable state. Intersections of 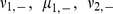 and 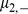 determine 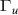. 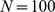, 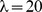, 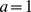, 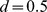.(EPS)

Text S1 **Mathematical Details.** The proof that (26) and (27) can be valid simultaneously.
(PDF)
