# Supplementary material for: Diffusive Public Goods and Coexistence of Cooperators and Cheaters on a 1D Lattice
Source: PLoS One. 2014 Jul 15;9(7):e100769. doi: 10.1371/journal.pone.0100769 (PMC4098918; doi:10.1371/journal.pone.0100769)
Supplement: Text S1 — Mathematical Details. The proof that (26) and (27) can be valid simultaneously. (PDF) [file pone.0100769.s002.pdf]

Supporting information

Let us study the first relation of (26) in a case when n_2 is fixed. So n_2 is considered as a parameter and let ν_1 be the extension of n_1 to positive real numbers. Knowing that $l = N - n_1 - n_2$ and let $x = e^{\nu_1/\lambda}$, $s = 1/(1-e^{-1/\lambda})$ and $b(n_2) = S_{n_2} e^{-(N-n_2-1)/\lambda}$, then I am looking forward the solutions of

$$s - s\frac{1}{x} - b(n_2)x = d^*, \tag{1}$$

to find n_1 values where the first relation of (26) is valid. It follows from (1) that

$$\nu_{1,\pm}(n_2) = \lambda log \left[\frac{(s-d^*) \pm \sqrt{(s-d^*)^2 - 4sb(n_2)}}{2b(n_2)} \right]. \tag{2}$$

ν_1 must be greater than two, since $n_1 = 2$ is the smallest possible P array in equilibrium thus
$\lambda log \left[\frac{(s-d^*)+\sqrt{(s-d^*)^2-4sb(n_2)}}{2b} \right] \geq 2$ must be valid. Consequently the first relation of (26) is satisfied if $2 \leq n_1 < \nu_{1,-}(n_2)$ or $n_1 > \nu_{1,+}(n_2) \geq 2$. The second relation of (26) can be analyzed similarly, that is n_2 is fixed and μ_1 is the extension of n_1 to positive real values. Using the same transformation and notations for the new parameters, I look for $y = e^{\mu_1/\lambda}$ which satisfies

$$s - se^{1/\lambda}\frac{1}{y} - b(n_2)e^{-1/\lambda}y = d^*. \tag{3}$$

Which leads the solutions of

$$\mu_{1,\pm}(n_2) = \lambda log \left[\frac{(s-d^*) \pm \sqrt{(s-d^*)^2 - 4sb(n_2)}}{2b(n_2)e^{-1/\lambda}} \right]. \tag{4}$$

Again $y_+ \geq e^{2/\lambda}$ must be true, that is $\lambda log \left[\frac{(s-d^*)\pm\sqrt{(s-d^*)^2-4sb(n_2)}}{2b(n_2)e^{-1/\lambda}}\right] \geq 2$ Thus the second relation of (26) is satisfied if $2 \leq \mu_{1,-} \leq n_1 \leq \mu_{1,+}$.

Comparing (33) and (35) it is clear that $\nu_{1,-}(n_2) < \mu_{1,-}(n_2)$ and $\nu_{1,+}(n_2) < \mu_{1,+}(n_2)$. It follows from these relations that if there is an integer n_1^* such that $2 \leq \nu_{1,+}(n_2) < n_1^* < \mu_{1,+}(n_2)$ then neither P nor NP spreads if the size of the array is n_1^*. If $\nu_{1,+}(n_2)$ and $\mu_{1,+}(n_2)$ do not straddle an integer, then the closest integers $n_1^{*,1} < \nu_{1,+}(n_2) < n_1^{*,2}$ $(n_1^{*,1} + 1 = n_1^{*,2})$ which straddle them form a stable state. Since at $n_1^{*,1}$ P spreads and NP does not spread, P array increases with a cell, thus $n_1^{*,1} \rightarrow n_1^{*,2}$ after this update. At this state however, NP spreads and P doesn't spread, thus system flips back to $n_1^{*,1}$ state. This is a locally stable cycle with two states. Similarly, if there is an integer n_1^{**} such that $2 \leq \nu_{1,-}(n_2) < n_1^{**} < \mu_{1,-}(n_2)$ then both P and NP spreads at n_1^{**}. So n_1^*-s define locally stable and n_1^{**}-s define locally unstable states.(Naturally n_1^* and n_1^{**} depend on n_2). If no integer straddled by $\nu_{1,-}(n_2)$ and $y_{1,-}(n_2)$ then P spreads and NP does not spread from $n_1^{**,1}$ while NP spreads and P doesn't spread from $n_1^{**,2}$ $(n_1^{**,1} < \nu_{1,-}(n_2) < \mu_{1,-}(n_2) < n_1^{**,2})$. This state is unstable.

The left hand side meeting point of P and NP arrays was analyzed in function of n_2. A completely similar calculation is applies to for the right hand side meeting point of P and NP arrays. Then I study the relations of (27) while n_1 is kept constant. Using the notations of $z = e^{\nu_2/\lambda}$ and $w = e^{\mu_2/\lambda}$

and following the analogous computations I find that

$$\nu_{2,\pm}(n_1) = \lambda log\left[\frac{(s-d^*) \pm \sqrt{(s-d^*)^2 - 4ab(n_1)}}{2b(n_1)}\right]$$
$$\mu_{2,\pm}(n_1) = \lambda log\left[\frac{(s-d^*) \pm \sqrt{(s-d^*)^2 - 4ab(n_1)}}{2b(n_1)e^{-1/\lambda}}\right],$$

where $b(n_1) = S_{n_1}e^{-(N-n_1-1)/\lambda}$.

Now I consider the continuous extensions of n_2 and n_1 of function b. Thus $\nu_{1+}(\nu_2)$ and $\nu_{2,+}(\nu_1)$ are continuous and strictly monotonously decreasing functions of ν_2 and ν_1, so there is at most one intersection of these functions. Because of symmetry in relations of (26) and (27) it is easy to see that this intersection always exists and here $\nu_{1,+} = \nu_{2,+}$. (Knowing the symmetry I can give the intersection point explicitly, but this is not essential in the analysis.) Let us denote the intersection point with $\{X_+^{(1)}, X_+^{(1)}\}$. Similarly the intersection of the continuous extensions of $\mu_{1,+}(\nu_2)$ and $\mu_{2,+}(\nu_1)$ functions is at a point where $\mu_{1,+} = \mu_{2,+}$, which is denoted by $\{Y_+^{(1)}, Y_+^{(1)}\}$. It follows from (33, 35, 5) that $X_+^{(1)} < Y_+^{(1)}$. Similarly we can determine the intersections of $\mu_{1,+}$ and $\nu_{2,+}$ and $\nu_{1,+}$ and $\mu_{2,+}$. Because of monotonicity, these functions also intersect only once. Denote these intersection points with $\{Y_+^{(2)}, Z_+^{(2)}\}$ and with $\{X_+^{(2)}, W_+^{(2)}\}$. Since $x_+ < y_+$ and $z_+ < w_+$ thus $\{Y_+^{(2)}, Z_+^{(2)}\}$, $\{X_+^{(1)}, X_+^{(1)}\}$,$\{X_+^{(2)}, W_+^{(2)}\}$ and $\{Y_+^{(1)}, Y_+^{(1)}\}$ are the vertices of a closed shape (Γ_s) bordered by the curves determined by functions $\nu_{1,+}, \mu_{1,+}, \nu_{2,+}$ and $\mu_{2,+}$. Every state is locally stable within this shape, that is neither P nor NP could spreads for every state $\{n_1^*, n_2^*\}\epsilon\Gamma_s$ (Fig. S1 a.). If there is no $\{n_1^*, n_2^*\}$ integer pair within Γ_s then the closest four integer points which straddle Γ_s

is a stable state (Fig. S1 b.). Since the location of update events is random, the motion is not periodic among these points.

Similarly we can consider the continuous extension of $\nu_{1,-}, \mu_{1,-}, \nu_{2,-}$ and $\mu_{2,-}$. These functions are strictly monotonously increasing in ν_1 and ν_2 (the continuous extensions of n_1 and n_2), thus $\nu_{1,-}, \mu_{1,-}, \nu_{2,-}$ and $\mu_{2,-}$ intersect in four points which determine a shape Γ_u where every state $\{n_1^{**}, n_2^{**}\}\epsilon\Gamma_u$ is unstable such that P and NP spread on both borders of P and NP arrays (Fig. S1 c).

The stable and unstable curves ($\nu_{i,-}, \nu_{i,+}$ and $\mu_{i,-}, \mu_{i,+}, i = 1, 2$) are connected at the points where $\nu_{i,-} = \nu_{i,+}$ and $\mu_{i,-} = \mu_{i,+}$. The complete phase space of the dynamics and the direction of motion are depicted in Figure S1 a. I note here that the phase space presented in Figure S1 differs from the standard phase space of differential equations, since the line pairs in Figure S1 determine stability or instability of left and right meeting points of P and NP arrays against the invasion of P and NP cells on the borders of arrays.
